# Correlation Between Transient Elastography and Non-invasive Biomarker Scores for the Detection of Liver Fibrosis

**DOI:** 10.7759/cureus.72892

**Published:** 2024-11-02

**Authors:** Muhammad Asif, Muhammad Sohaib, Waleed Anwaar, Adeel Ahmed, Neha Tehreem Khalid, Hanzala Tariq, Muhammad Irfan Jamil

**Affiliations:** 1 Medicine, Jinnah Hospital, Lahore, PAK; 2 Medicine, Nishtar Medical University, Multan, PAK; 3 Medicine, Lahore General Hospital, Lahore, PAK; 4 Gastroenterology and Hepatology, Bahawal Victoria Hospital, Bahawalpur, PAK; 5 Health, Quaid-e-Azam Medical College, Bahawalpur, PAK; 6 Nephrology, Lahore General Hospital, Lahore, PAK

**Keywords:** • chronic liver disease, fibrosis, hepatitis c virus (hcv), non-invasive biomarkers, transient elastography (te)

## Abstract

Background and aim

The study aimed to address the need for reliable and non-invasive biomarkers (NIBM) for detecting fibrosis among patients with chronic liver disease (CLD).

Material and methods

This was a diagnostic validation study executed at the Department of Gastroenterology, Jinnah Hospital, Lahore. The study was carried out from July 2023 to June 2024, enrolling a total of 88 patients using non-probability consecutive sampling. Patients with chronic liver disease (CLD) due to chronic viral hepatitis B (HBV), hepatitis C (HCV), and non-alcoholic fatty liver disease (NAFLD) were included in the study. A cut-off value of 12.5 kPa was used to label fibrosis using transient elastography. Blood samples were collected for recording values of alanine aminotransferase (ALT), aspartate aminotransferase (AST), and platelet count, and the appropriate formulas were applied to calculate the aspartate aminotransferase to platelet ratio index (APRI), Fibrosis-4 (FIB-4), AST/ALT-to-platelet ratio index (AARPRI), and BARD scores (a scoring system for NAFLD fibrosis that predicts the risk of advanced fibrosis in NAFLD patients; the components include body mass index (BMI), aspartate aminotransferase (AST)/alanine aminotransferase (ALT) ratio, and diabetes status). Spearman's rank correlation coefficient was used to assess the strength and direction of the association between these variables.

Results

Among the 88 patients, 61.4% were male and HCV was the most common cause of CLD (63.6%). Statistically significant correlations were found between transient elastography and NIBM: FIB-4 (p < 0.001, area under receiver operating characteristic curves (AUROC), 0.835; sensitivity, 47.7%; specificity, 90.9%), APRI (p = 0.020; AUROC, 0.769; sensitivity, 25.0%; specificity, 93.2%), AARPRI (p = 0.011, AUROC, 0.782), and BARD (p = 0.033; AUROC, 0.648). FIB-4 demonstrated the strongest correlation with liver stiffness measurements (LSM) (r = 0.617), indicating its reliability in detecting liver fibrosis, whereas the aspartate aminotransferase to alanine aminotransferase ratio (AAR) showed the weakest correlation (r = 0.163).

Conclusion

This study reported that FIB-4 displayed the highest correlation with liver stiffness measurements obtained through transient elastography in detecting liver fibrosis. APRI and AARPRI reported a moderate correlation, while AAR and BARD were less consistent in their performance. These findings suggest that FIB-4, APRI, and AARPRI are effective non-invasive tools for assessing liver fibrosis in a limited resource setting where transient elastography is not available.

## Introduction

Liver fibrosis remains an essential hallmark in the progression of chronic liver diseases, ultimately leading to cirrhosis and hepatocellular carcinoma (HCC) [1]. Viral hepatitis B and C are the primary causes of liver fibrosis in Asia, whereas non-alcoholic fatty liver disease is the leading cause in Western countries [2]. Epidemiological studies indicate that 18 million people in Pakistan are affected by viral hepatitis B and C infections [3].

Liver biopsy has been employed for the evaluation of liver histology and the progression of chronic liver diseases [4]. However, it is invasive and carries risks of complications, ranging from abdominal pain to hemorrhage and biliary system injury. These potential complications have led some patients to decline liver biopsy as a diagnostic option [5]. Alternatively, transient elastography emerges as a non-invasive imaging modality that utilizes ultrasound (USG) and low-frequency elastic waves to measure the liver stiffness and determine the stages of liver fibrosis [6]. In the realm of non-invasive liver fibrosis assessment, Lemoine and colleagues conducted a study that substantiated the value of aspartate transaminase-AST to platelet ratio index (APRI) and FIB-4 scores. Their research revealed the area under the receiver operating characteristic curves (AUROCs) of 0.86 and 0.81, respectively, when compared to the liver biopsy as a gold standard [7].

Given the invasiveness and complications associated with liver biopsy and limited availability of transient elastography, there is a need to validate non-invasive biomarkers for fibrosis assessment in low-resource healthcare settings [8]. The study aimed to determine the diagnostic accuracy of FIB-4, APRI, AARPRI, and BARD scores in comparison to transient elastography so as to provide safer and more accessible alternatives for fibrosis (liver elasticity and viscosity) staging in patients with chronic liver disease (CLD).

## Materials and methods

A diagnostic validation study was conducted at the Department of Gastroenterology, Jinnah Hospital, Lahore over a period of 12 months from July 2023 to June 2024. Ethical approval was obtained from the institutional review board (IRB # 379/20/10/2023/S1 ERB) and informed consent from the patients in accordance with the Helsinki declaration. A total of 88 patients were included using non-probability consecutive sampling based on the expected sensitivity (77%) and specificity (79%) of FIB-4 for diagnosing liver fibrosis [9]. Both female or male patients aged 20-70 years with HBV/HCV infection or NAFLD-related chronic liver disease were included. Patients diagnosed with portal vein thrombosis (PVT), hepatic vein thrombosis, hepatocellular carcinoma (HCC), autoimmune hepatitis, primary biliary cirrhosis (PBC), and Wilson's disease and congestive heart failure were also excluded. Patients with a history of immunosuppressant use that may alter fibrosis progression were also excluded. A structured questionnaire was administered to collect the demographic information (age, gender, BMI), medical history (presence of HCV/HBV infection, NAFLD), and clinical data (duration of liver disease, any comorbidities). Blood samples were collected from participants to measure aspartate aminotransferase, alanine aminotransferase, and platelet count. These laboratory results were utilized to calculate non-invasive biomarker scores (aspartate aminotransferase to platelet ratio index (APRI), fibrosis-4 (FIB-4), aspartate to alanine aminotrasferase ratio (AAR), AST/ALT-to-platelet ratio index (AARPRI), BARD [a scoring system for NAFLD fibrosis that predicts the risk of advanced fibrosis in NAFLD patients. The components include body mass index (BMI), aspartate aminotransferase (AST)/alanine aminotransferase (ALT) ratio, and diabetes status]) using their respective formulae. The diagnostic cut-offs for liver fibrosis were APRI > 2.0, FIB-4 > 3.5, AAR ≥ 0.9, AARPRI ≥ 0.8, and BARD score ≥ 3. Transient elastography was performed by a trained operator, adhering to the standard protocol. Liver stiffness measurements (LSM) were assessed in kilopascals (kPa), with cut-off value of 12.5 kPa used to determine the presence of liver fibrosis. Liver stiffness correlates with fibrosis: the stiffer the liver, the more advanced the fibrosis.

Data was analyzed using SPSS software version 26.0 (IBM Corp., Armonk, NY, USA). Numerical variables were expressed in the form of mean±standard deviation. For qualitative variables, frequencies and percentages were calculated. The diagnostic accuracy of each non-invasive biomarker score of liver fibrosis was calculated using 2×2 tables constructed taking transient elastography as standard. Furthermore, Spearman's rank correlation coefficient was evaluated to analyze the degree of association between transient elastography and NIBMs. The performances of non-invasive biomarker scores (NIBM) in diagnosing cirrhosis were evaluated using the AUROCs. The AUROC calculations were performed using the binomial method, with transient elastography considered as the gold standard for comparison. A P-value of <0.05 was considered statistically significant.

## Results

This study included a total of 88 patients, with a majority being male (54; 61.4%). The most common etiology of CLD was HCV infection, affecting 56 patients (63.6%), followed by HBV infection in 23 patients (26.1%) and NAFLD in 10.2% (Table 1).

**Table 1:** Baseline Characteristics of the Study Population BMI: Body mass index; ALT: alanine aminotransferase; AST: aspartate aminotransferase; LSM: liver stiffness measurement.

Descriptive Statistics	Range	Mean± Std. Deviation
Age	48	46.82±11.74
BMI	13.9	27.414±3.71
Duration of Chronic Liver Disease	6	4.39±1.24
ALT Level	69	65.99±18.59
AST Level	90	58.17±20.61
Platelets	240	172.75±57.15
LSM Measurement	16.5	11.289±4.37

Table 2 reported the diagnostic agreement between non-invasive biomarkers and transient elastography for detecting liver fibrosis. The liver fibrosis was diagnosed in 44 (50%) of patients using transient elastography, with a cut-off value of more than 12.5 kPa.

**Table 2:** Contingency Tables for Non-Invasive Biomarkers Compared with Transient Elastography for Diagnosing Liver Fibrosis APRI: Aspartate aminotransferase to platelet ratio index; FIB-4: Fibrosis-4; AAR: aspartate to alanine aminotransferase ratio; AARPRI: AAR platelet ratio index; LSM: liver stiffness measurement. Chi-square statistical test is used, taking p-value of less than 0.05 as significant.

FIB-4 Diagnosis		Transient Elastography Positive	Transient Elastography Negative	Chi-square (p-value)
FIB-4	Positive	21	4	16.147 (0.001)
	Negative	23	40	
APRI	Positive	11	3	5.436 (0.020)
	Negative	33	41	
AAR	Positive	40	39	0.124 (0.725)
	Negative	4	5	
AARPRI	Positive	39	29	6.471 (0.011)
	Negative	5	15	
BARD	Positive	28	18	4.555 (0.033)
	Negative	16	26	

FIB-4 demonstrated the highest diagnostic accuracy (69.3%) with good sensitivity (47.7%) and specificity (90.9%), along with a robust AUROC of 0.835 (95% CI: 0.751-0.920, p < .001), indicating excellent diagnostic performance. AARPRI followed with an AUROC of 0.782 (95% CI: 0.684-0.880, p < .001) and a diagnostic accuracy of 61.4%. APRI showed high specificity (93.2%) but low sensitivity (25.0%) and AUROC of 0.769 (95% CI: 0.668-0.871, p < .001). AAR displayed the highest sensitivity (90.9%) but the lowest specificity (11.4%), diagnostic accuracy (51.1%) and AUROC of 0.614 (95% CI: 0.496-0.733, p = .065), indicating poor diagnostic reliability. BARD showed moderate performance across all parameters with a AUROC of 0.648 (95% CI: 0.533-0.762, p = 0.017). FIB-4 also showed the strongest correlation with liver stiffness measurements (r = 0.617, p < 0.001), while AAR had the weakest correlation (r = 0.163, p = .129). These findings suggest that FIB-4 and AARPRI are reliable non-invasive tools for assessing liver fibrosis in clinical practice (Table 3).

**Table 3:** Diagnostic Performance of Non-Invasive Biomarkers for Liver Fibrosis Assessment P-values indicate the significance of Spearman's correlation coefficients. Abbreviations: APRI: Aspartate aminotransferase to platelet ratio index; FIB-4: Fibrosis-4; AAR: aspartate to alanine aminotransferase ratio; AARPRI: AAR Platelet Ratio Index; LSM: liver stiffness measurement; PPV: positive predictive value; NPV: negative predictive value.

Biomarker	Sensitivity	Specificity	PPV	NPV	Diagnostic Accuracy	AUROC	Correlation Coefficient with LSM	P-value
APRI	25.00%	93.20%	78.60%	55.40%	59.10%	0.769	0.552	0.001
FIB-4	47.70%	90.90%	84.00%	63.50%	69.30%	0.835	0.617	0.001
AAR	90.90%	11.40%	50.60%	55.60%	51.10%	0.614	0.163	0.129
AARPRI	88.60%	34.10%	57.40%	75.00%	61.40%	0.782	0.506	0.001
BARD	63.60%	59.10%	60.90%	61.90%	61.40%	0.648	0.224	0.036

## Discussion

FIB-4 emerged as the strongest performer in our study with an AUROC of 0.835, suggesting excellent diagnostic ability. The positive predictive value (PPV) of 84%, the highest among the evaluated biomarkers, confirms that a positive FIB-4 result is very likely to reflect true fibrosis. The diagnostic accuracy of FIB-4 was at 69.3%. APRI is highly effective in correctly identifying patients without fibrosis. The diagnostic accuracy of APRI was the lowest among the biomarkers at 59.1%, suggesting that while it has a role in fibrosis detection, it should not be used in isolation. AAR's exceptionally high sensitivity of 90.9% indicates that it is highly likely to detect fibrosis when present; however, its specificity of 11.4% is notably low, suggesting a high rate of false-positive results. The PPV of 50.6% and NPV of 55.6% reveal that AAR may not be the most reliable indicator of actual fibrosis status, and its diagnostic accuracy of 51.1% indicates that more than half of the time, the results can be trusted to align with transient elastography findings. AARPRI, with a sensitivity of 88.6%, positions itself as a strong screening tool. Its specificity of 34.1% is modest, suggesting that while it's good at identifying those with the condition, it may also falsely categorize healthy individuals as having fibrosis. The diagnostic accuracy of 61.4% demonstrates that it is a fairly reliable biomarker. The BARD score, with a sensitivity of 63.6% and specificity of 59.1%, has a balanced diagnostic profile. The diagnostic accuracy of 61.4% puts it on par with AARPRI.

When examining the correlation coefficients, the strong correlation of FIB-4 (r = 0.617) with LSM validates its utility in clinical settings. The significant correlations for APRI (r = .552) and AARPRI (r = 0.506) with LSM also support their use as potential tools for fibrosis assessment, though they are not as robust as FIB-4. The AAR score has a weaker correlation (r = .163) with LSM. In light of the AUC values, FIB-4's dominance is clear, suggesting that it has the best discriminatory power among the biomarkers tested. The AUC for APRI and AARPRI, while lower than FIB-4, still indicates good predictive ability. The findings suggest a hierarchy in the reliability of NIBMs, led by FIB-4 and followed by APRI, AARPRI, and BARD, with AAR having the least diagnostic utility.

A meta-analysis (2017) of 13,046 NAFLD patients found that APRI and FIB-4 are effective in diagnosing advanced fibrosis, with APRI showing sensitivities of 50.0% and 18.3% at thresholds of 1.0 and 1.5, and specificities of 84.0% and 96.1%. FIB-4's sensitivities were 26.6% and 31.8% at cutoffs of 2.67 and 3.25, with specificities of 96.5% and 96.0%. These results particularly for FIB-4 align with our study's findings, underscoring FIB-4’s consistent diagnostic reliability [10].

Hussain et al. (2019) reported a predictive capacity for APRI and FIB-4, with AUC values indicating good predictive ability for these biomarkers of fibrosis stages in patients with HCV. The study noted an AUC of 0.864 for APRI when predicting cirrhosis (F4), which underlines the biomarker’s effectiveness. Similarly, the FIB-4 index with a cutoff value of more than 3.25 for stage F4 demonstrated a specificity of 72.3% and a sensitivity of 53.2%, highlighting its diagnostic potential [11]. In Peleg et al.'s study (2017), APRI and FIB-4 were evaluated for predicting fibrosis in NAFLD and chronic HCV patients. APRI showed an AUC of 0.8307 for NAFLD, less effective than for HCV (AUC 0.9965), and inferior to FIB-4’s AUC of 0.8959. This mirrors our findings, highlighting FIB-4's broader applicability and APRI's varying efficacy across liver diseases [12].

Taneja et al. (2016) reported a high AUROC of 0.896 for FIB-4 with a positive ratio of 13.4, indicating its strong diagnostic precision for cirrhosis. This outperformed the APRI score, which showed a lower AUROC of 0.823 and a moderate positive likelihood ratio of 6.9 [13]. Yunihastuti et al.'s retrospective analysis revealed that an APRI threshold of 1 had a specificity of 95%, a sensitivity of 48.4%, and correctly classified 81.6% of the patients, resulting in a moderate AUC of 0.72. The FIB-4 index, with a cutoff of 1.66, demonstrated a specificity of 92.5%, a sensitivity of 53.1%, and accurately classified 81.1% of the patients, with an AUC of 0.73. These findings suggest that both APRI and FIB-4 have moderate efficacy with a high specificity in diagnosing cirrhosis [14]. The high specificity of both markers aligns well with our findings, underscoring their potential role in the clinical assessment of liver fibrosis and cirrhosis. Zhu et al. (2011) found that APRI had an AUROC of 0.81 for significant fibrosis and 0.83 for cirrhosis in chronic HBV patients. FIB-4 demonstrated a slightly higher AUROC of 0.86 for significant fibrosis but a lower AUROC of 0.77 for cirrhosis [15]. Wai et al. (2003) noted that in the chronic hepatitis C patients, APRI had an AUC of 0.80 for significant fibrosis and 0.89 for cirrhosis in the training set, increasing to 0.88 and 0.94 in the validation set. This indicates a high reliability of APRI in predicting cirrhosis, particularly when validated against a separate cohort [16]. Chowdhury et al. (2017) noted that in their study cohort, FIB-4 showed a moderate positive correlation with TE, while APRI demonstrated a weaker correlation. They reported that FIB-4 was a good predictor of liver fibrosis with 72% diagnostic accuracy when using TE as the gold standard. This aligns with our study's findings [17].

Limitations

This study offers valuable insights into the diagnostic accuracy of non-invasive biomarkers (NIBM) for liver fibrosis with certain limitations. The use of non-probability convenient sampling, while efficient, may limit the generalizability of our findings across diverse patient populations. These biomarkers and transient elastography (TE) have limitations in detecting early-stage fibrosis, especially in populations like obese individuals, warranting complementary clinical assessment in decision-making. Additionally, the single-center design restricts the validity of the results. However, the study's strengths lie in its systematic approach, including the use of chi-square tests, 2x2 tables, AUROC evaluations, and Spearman's correlation analysis, which enhance the reliability of our findings. Future studies should consider multicenter designs and a diverse population to extend the applicability of the results. Exploring these biomarkers in different liver pathologies and in longitudinal settings could provide deeper insights into their utility in monitoring disease progression and response to therapy.

## Conclusions

The findings of the study indicate that FIB-4 is the most reliable non-invasive biomarker for detecting liver fibrosis, as it demonstrates a strong correlation with liver stiffness measurements obtained through transient elastography. APRI and AARPRI also exhibited substantial diagnostic performance, supporting their use as alternative non-invasive methods for the evaluation of liver fibrosis. In contrast, AAR and BARD showed limited diagnostic accuracy and a weaker correlation with liver stiffness. These results highlight the potential clinical application of FIB-4, APRI, and AARPRI in the non-invasive assessment of liver fibrosis, especially in settings where transient elastography is not readily available.
